# Elevated white blood cell counts in ischemic stroke patients are associated with increased mortality and new vascular events

**DOI:** 10.3389/fneur.2023.1232557

**Published:** 2023-09-12

**Authors:** Thao Phuong Vo, Marie Hvelplund Kristiansen, Hans Carl Hasselbalch, Troels Wienecke

**Affiliations:** ^1^Neurology Department, Zealand University Hospital, University of Copenhagen, Roskilde, Denmark; ^2^Hematology Department, Zealand University Hospital, University of Copenhagen, Roskilde, Denmark

**Keywords:** WBC (leukocyte), mortality, vascular event, ischemic stroke, 10 year follow up

## Abstract

**Background and purpose:**

High levels of white blood cells (WBC) in ischemic stroke have been shown to increase the risk of new vascular events and mortality in short and intermediate follow-up studies, but long-term effects remain unknown. We studied whether elevated levels of WBC in ischemic stroke patients are associated with new vascular events and mortality in a 10-year follow-up period.

**Methods:**

We included ischemic stroke patients hospitalized between 2011 and 2012, categorizing their WBC counts within 48 h of stroke onset as high or normal (3.5–8.8 × 10^9^ mmol/L; >8.8 × 10^9^ mmol/L). Using Aahlen Johansen and Cox proportional hazard models with competing risk, we analyzed the association between WBC levels and new vascular events. Kaplan–Meier and standard Cox proportional hazard models were used to assess the risk of all-cause mortality.

**Results:**

Among 395 patients (median age 69, [IQR: 63, 78], female patients 38,0%), 38.5% had elevated WBC at admission. During the 10-year follow-up, 113 vascular events occurred, with 46% in patients with elevated WBC and 54% in patients with normal WBC. After adjusting for relevant factors, elevated WBC levels were independently associated with increased risk of new vascular events (HR: 1.61, CI: 1.09–2.39 *p* < 0.05) and death (HR: 1.55, CI: 1.15–2.09, *p* < 0.05).

**Conclusion:**

Elevated WBC levels in ischemic stroke patients are linked to a higher risk of new vascular events and mortality. Thus, ischemic stroke patients with elevated WBC without clinical infection need special attention to investigate possible underlying conditions to prevent future vascular events and reduce mortality. The interpretation of our results is limited by the absence of adjustment to premorbid functional status, stroke severity, and stroke treatment.

## Introduction

Stroke is the second leading cause of death worldwide (1), with a 1-month case fatality of 13.5% (2). Even for patients who survive the 1st months of recovery, the long-term mortality and morbidity rates remain persistently high (3). Alongside the high mortality rates, patients with ischemic stroke carry an increased risk of recurrent stroke despite optimal preventive treatment. According to a Danish nationwide registry study, the risk of stroke recurrence is estimated to be 4, 10, and 13% for the 1-, 5-, and 10-year risks, respectively (4). A recurrent stroke is proving often more lethal and disabling than a first stroke (3, 5, 6). Although causes of recurrent stroke are still largely unknown, inflammation may be a major risk factor, as both acute and chronic inflammation contribute to atherogenesis and thereby the formation of thrombosis (7).

Biomarkers of inflammation such as white blood cells (WBC) have been associated with an increased risk of new ischemic events and death (8–15); however, when analyzing the risk of recurrent stroke, previous studies did not explicitly account for the competing risk of death (9–12) and the presence of infection at admission was not consistently adjusted for, potentially producing a significant bias. Finally, the majority of studies were registry-based and had limited follow-up time, with none of them having a follow-up period longer than 5 years.

This study aimed to evaluate the association between elevated levels of WBC and the rate of a new vascular event and death by manually reviewing 395 patient records over a 10-year follow-up period taking both infection and competing risk of death into consideration.

## Methods

### Study population

This retrospective, observational study was conducted on ischemic stroke patients admitted at the Department of Neurology, Zealand University Hospital, Roskilde, Denmark, between September 2011 and September 2012. Follow-up was obtained from October 2021 to May 2022.

Patients were included if blood samples were collected within 48 h of symptom onset. The stroke diagnoses were established by a stroke neurologist and the plausibility of the diagnosis was further assessed during data collection by excluding patients who were later diagnosed with a more conceivable disease causing the neurological deficits.

Data regarding laboratory results, sex, medical history, medicine, lifestyle, a new vascular event, and death were collected through a manual review of the electronic patient medical files. Electronic patient medical files contain all existing medical records and paraclinical examinations conducted on patients in hospitals in Denmark. Hypertension, hyperlipidemia, and diabetes were registered if patients received corresponding medicine at admission or if the diseases were reported in the records. Atrial fibrillation was registered if confirmed from ECG during hospitalization, 7 days continuous ECG monitoring, or if previously recorded. Additionally, infection was registered if antibiotics were prescribed during the first 2 days of hospitalization. Overweight was determined through either a body mass index (BMI) exceeding 25 kg/m^2^ or reported by the evaluating physician as BMI was inconsistently registered. Previous vascular events included transient ischemic attack, acute myocardial infarction (AMI), ischemic stroke, retinal artery occlusion, gastrointestinal embolism, pulmonary embolism (PE), and deep vein thrombosis (DVT).

WBC levels were divided into normal or elevated according to the cutoff values made by the Central Lab which were as follows: 3.5–8.8 × 10^9^ mmol/L; > 8.8 × 10^9^ mmol/L.

### Outcome

The primary outcome of this study was the occurrence of a new vascular event. A new vascular event involved recurrent ischemic stroke, DVT, PE, retinal artery occlusion, peripheral arterial thrombosis, gastrointestinal thrombosis, or AMI. All patients’ discharge letters were screened for new events. To minimize underestimation of a new vascular event, laboratory results were systematically reviewed for elevated D-dimer and troponins (TnT and TnI) as they are an integrated part of the standard investigation in Denmark when suspecting, respectively, DVT or AMI. If elevated, patient journals were assessed to evaluate if the diagnosis was confirmed. Furthermore, computed tomography scans of the thorax and brain were systematically reviewed for suspicion of PE and brain infarction. The secondary outcome was all-cause mortality. All patients with residence in Denmark were followed until death or the day of data collection.

The study was approved by the hospital administration at Zealand University Hospital according to guidelines. The project was registered at the regional registry for data protection in Region Zealand (REG-111-2021). Data were handled in accordance with the Danish Data Protection Act and Regulation.

### Statistical analysis

Baseline characteristics of WBC were analyzed by Fisher’s exact test for categorical variables and the ANOVA test for continuous variables. A *p-*value of ≤0.05 was considered to be statistically significant.

Kaplan–Meier curves and log-rank tests were used to assess 1-, 5-, and 10-year mortality among WBC terciles. Aalen–Johansen and Gray’s tests were used to estimate new vascular events for the same subgroups and time points.

The Cox proportional hazards regression model was used to determine hazard ratios (HR) with a 95% confidence interval (CI) concerning death. The cause-specific Cox proportional hazards model was carried out to determine the association of WBC with a new vascular event with 95% CI. The proportional hazards assumption was tested by using Schoenfeld residuals, which showed no violation of the model (Supplementary Table 1). Possible confounders such as age, sex, overweight, diabetes, hypertension, hyperlipidemia, atrial fibrillation, previous vascular events, smoking status, and infection at admission were adjusted for in multivariate models. Sensitivity analysis was conducted on a subgroup with arterial events, and the analysis was also performed without considering competing risks.

Statistical analysis was performed using R studio, version 2022.02.0.

## Results

A total of 395 patients were included in this study. The median follow-up time was 9.90 years [IQR: 9.86, 10.0], the median age at baseline was 69 years [IQR: 63, 78], and 38.0% were women. The number of patients with elevated WBC at admission was 152 (38.5%). Baseline characteristics are presented in Table 1.

**Table 1:** Baseline characteristics, white blood cell.

	Overall (*n* = 395)	Normal (*n* = 243)	High (*n* = 152)	value of *p*
	*n* (%) or median [IQR]	*n* (%) or median [IQR]	*n* (%) or median [IQR]	
**Demography and clinical features**				
Age, median	69 [63, 78]	70.0 [64, 78]	68 [61, 77]	0.178
Female	150 (38.0)	87 (35.8)	63 (41.4)	0.287
**Medical history**				
Diabetes	43 (10.9)	20 (8.2)	23 (15.1)	**0.045**
Hypertension	144 (36.5)	85 (35.0)	59 (38.8)	0.454
Hyperlipidemia	54 (13.7)	29 (11.9)	25 (16.4)	0.229
Atrial fibrillation	125 (31.6)	77 (31.7)	48 (31.6)	1
Previous vascular event	108 (27.3)	60 (24.7)	48 (31.6)	0.164
Overweight	164 (41.5)	99 (40.7)	65 (42.8)	0.880
Normal	104 (26.3)	66 (27.2)	38 (25.0)	
Missing data	127 (32.2)	78 (32.1)	49 (32.2)	
Ever smoking	256 (64.8)	149 (61.3)	107 (70.4)	0.119
Never	117 (29.6)	81 (33.3)	36 (23.7)	
Missing data	22 (5.6)	13 (5.3)	9 (5.9)	
Ever alcohol	75 (19.0)	47 (19.3)	28 (18.4)	0.895
Never	294 (74.4)	181 (74.5)	113 (74.3)	
Missing data	26 (6.6)	15 (6.2)	11 (7.2)	
Infection at admission	82 (20.8)	36 (14.8)	46 (30.3)	**<0.001**

IQR, interquartile range; value of *p* comparing high levels with low levels of WBC.*P* values in bold are < 0.05.

Significantly more patients with elevated WBC had a history of diabetes compared to the patients without elevated WBC. Moreover, patients with elevated levels of WBC had a higher occurrence of infection at admission compared to patients without elevated levels of WBC (Table 1).

### New vascular event

**Figure 1:**
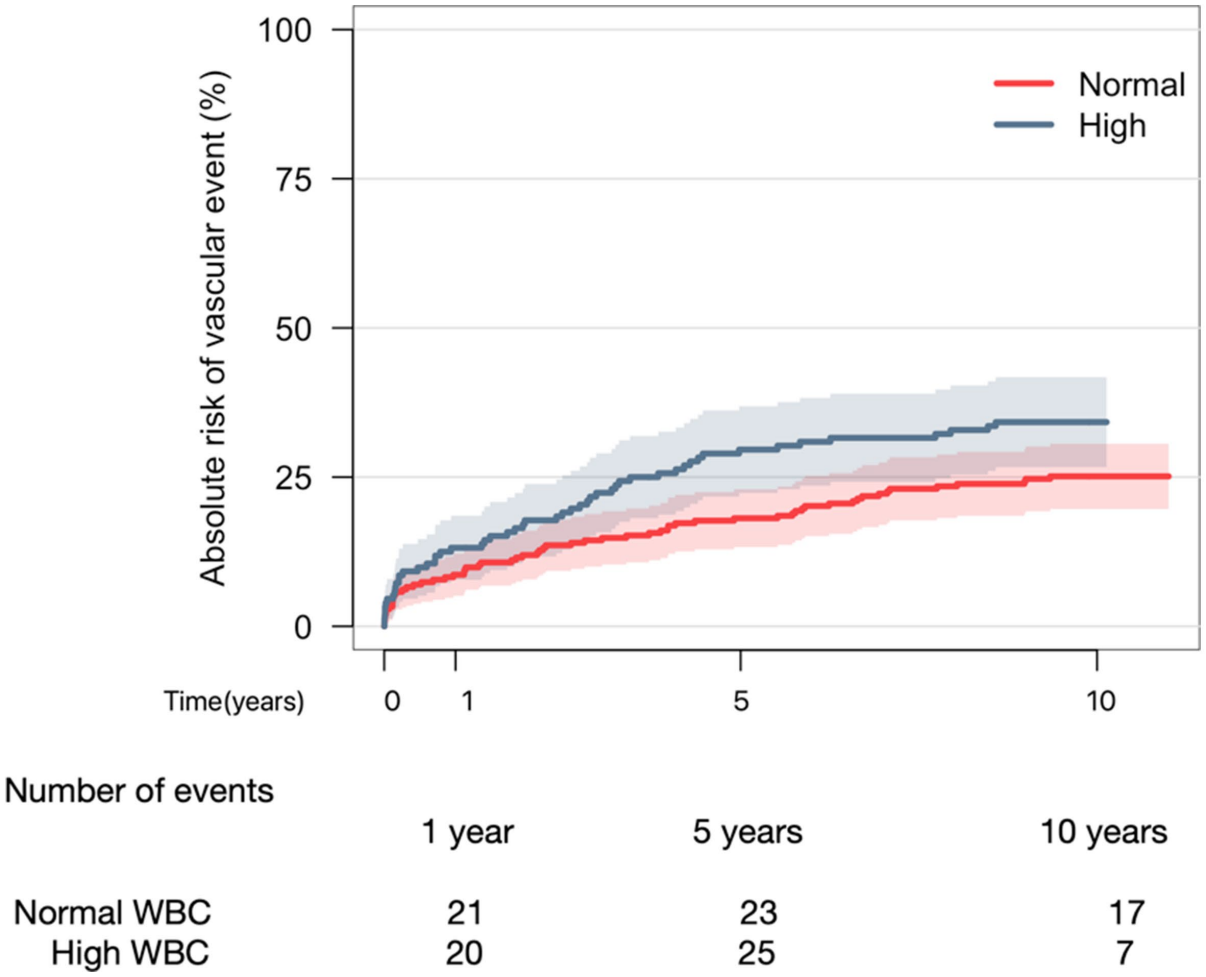
Aalen-Johansen curve depicting 10-year risk of vascular event when comparing normal WBC to elevated WBC. Gray’s test (*p*-value = 0.089).

Cumulative incidence of a new vascular event in patients with elevated levels of WBC was 13%, 30%, and 34% compared to 9%, 18%, and 25% in patients with normal levels of WBC after 1%, 5%, and 10 years, respectively (Figure 1). There was a trend toward more vascular events in patients with high levels of WBC, yet Gray’s test did not reveal a statistical difference between the two graphs (*p* = 0.089).

In univariate cause-specific Cox regression analyses, patients with elevated levels of WBC were at greater risk of a new vascular event compared to patients with normal levels (HR: 1.25, CI: 1.04–1.51, *p* < 0.05). Moreover, patients with previous vascular events (HR: 2.05, CI: 1.40–3.00, *p* < 0.05) were at greater risk of a new vascular event (Supplementary Table 3). After adjustment in multivariate analysis, elevated WBC was still associated with an increased risk of new vascular events (HR: 1.61, CI: 1.09–2.39, *p* < 0.05).

**Figure 2:**
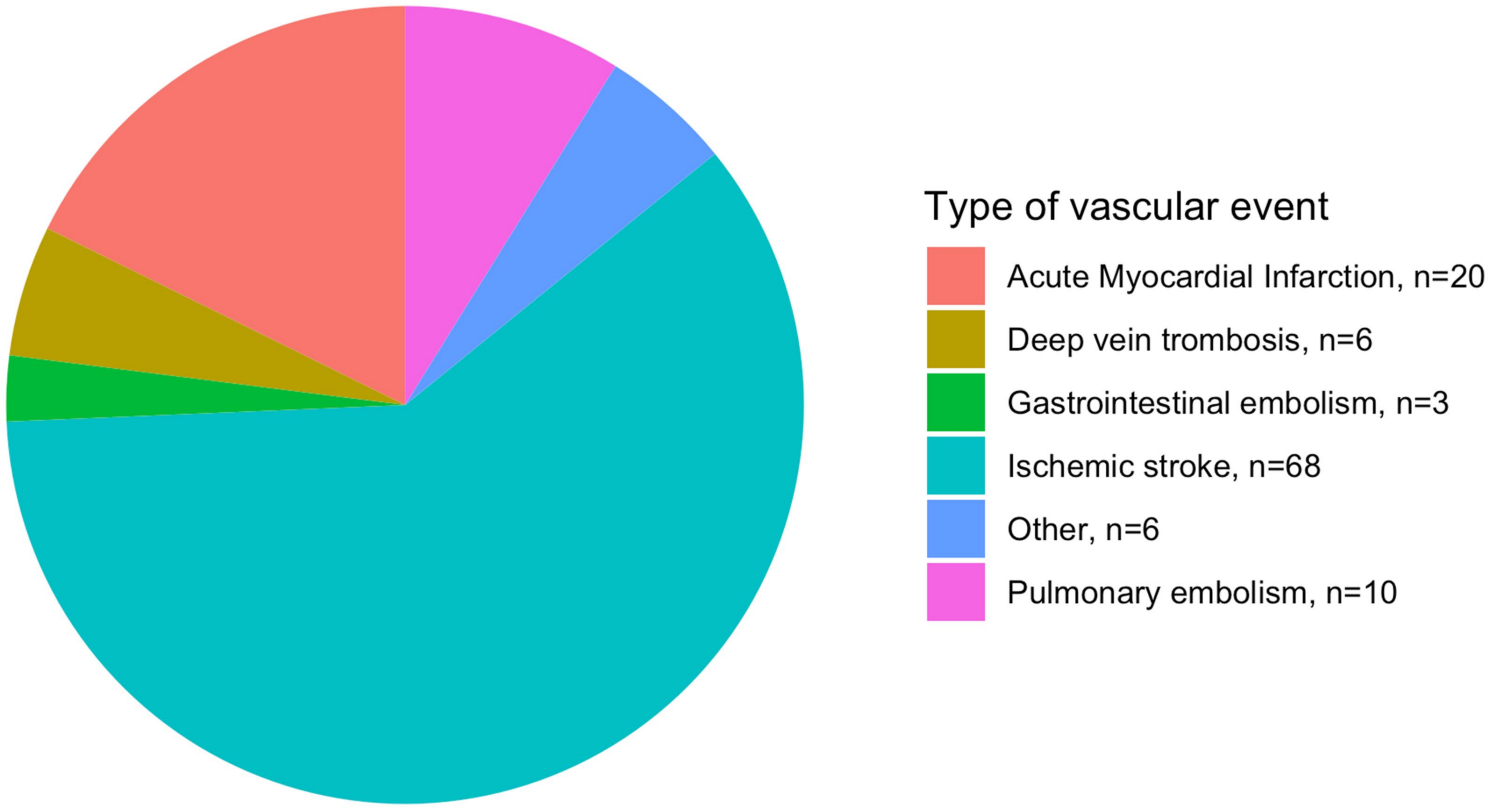
Distribution of new vascular events in patients with ischemic stroke. n, number of patients.

The distribution of new vascular events is illustrated in Figure 2. Sensitivity analyses with sub-analysis according to arterial events showed robustness (HR: 1.68, CI: 1.09–2.60, *p* = 0.019), while analysis without considering competing risks did not (Supplementary Table 2).

### Survival

**Figure 3:**
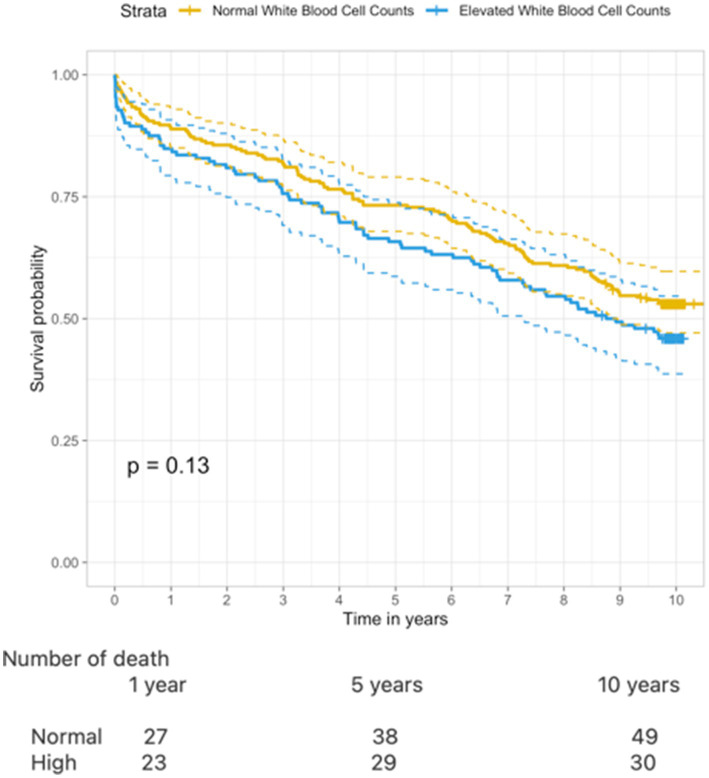
Kaplan–Meier curve illustrating 10–year survival when comparing normal WBC and elevated WBC (log rank test *p* = 0.13).

Overall survival probability in patients with high levels of WBC was 84%, 65%, and 46% at 1, 5, and 10 years, respectively, whereas patients with normal levels of WBC had 88% survival probability after 1 year, declining to 73% and 53% after 5 and 10 years (Figure 3). The cumulative incidence of death revealed a trend toward a higher risk of death when comparing patients with high levels of WBC to those with normal levels (log-rank *p* = 0.13).

Univariate Cox regression analysis showed that older age (HR: 1.10, CI: 1.09–1.12, *p* < 0.001), atrial fibrillation (HR: 1.78, CI: 1.34–2.37, *p* < 0.001), infection (HR: 1.99, CI: 1.46–2.72, *p* < 0.05), and previous vascular events (HR: 1.92, CI: 1.43–2.57, *p* < 0.05) were associated with an increased risk of all-cause mortality (Supplementary Table 4). After adjustment in multivariate Cox regression analysis, high levels of WBC were independently associated with an increased risk of death (HR: 1.55, CI: 1.15–2.09, *p* < 0.05).

## Discussion

In this study, with a follow-up of 10 years, we demonstrate that ischemic stroke patients with elevated levels of WBC have an increased risk of death and new vascular events. The association was present even after adjustment for possible confounding factors.

To the best of our knowledge, we are the first to evaluate the association between elevated levels of WBC and the risk of new vascular events and death by thoroughly reviewing patient records with a 10-year follow-up period taking both infection and competing risk of death into consideration. Prior studies that only partially assessed this association may have resulted in either overestimation or underestimation of risk. Despite differences in methods and design, we find that our results are in line with previous studies in the field. The vast majority of studies investigating WBC and new vascular events in ischemic stroke patients find a significant association (8, 10–12). A study of 18,558 participants demonstrated that patients with WBC in the top quartile (>8.2 × 10^9^/L) compared with the quartile with the lowest leukocyte counts at baseline (>5.9 × 10^9^/L) had higher risks of ischemic stroke (RR: 1.30; *p* = 0.007), myocardial infarction (RR: 1.56; *p* = 0.001), and vascular death (RR: 1.51; *p* = 0.001) (10). Only one study found no statistical correlation between elevated WBC and new ischemic events (9).

Similarly, multiple studies demonstrate that elevated levels of WBC significantly increase the risk of mortality (11–15). The largest cohort of 14,174 patients found a 1.7-fold increase in all-cause mortality for elevated levels of WBC (HR: 1.7, CI: 1.28–2.26) (12). However, direct comparison between studies is difficult as different statistical methods and study designs have been used. The risk of new vascular events has commonly been evaluated with the logistic regression or standard Cox proportional hazards model (9–12). Unlike cause-specific Cox proportional hazard regression, logistic regression produces a probability of event without integrating the aspect of “time-to-event,” and the standard Cox proportional hazards model does not account for competing risks. Only one study has explicitly reported the use of cause-specific Cox proportional hazard regression to assess the hazard of new vascular events; however, it did not adjust for other possible confounders. The study, however, censored patients upon death using Kaplan–Meier curves to evaluate the risk of new events (8). Since mortality is high following ischemic stroke, it should be considered a substantial competing risk. Therefore, the use of Kaplan–Meier violates the assumption of non-informative censoring, ultimately leading to a possible overestimation of risk, compared to an Aahlen Johansen model. Available studies make use of different study designs. For example, WBC counts were determined differently. Some studies measured values of WBC with continuously measured values (8, 11), while others compared categorical values with arbitrary cutoff points (10, 12–14). The varied definitions of elevated biomarkers can potentially cause an overestimation or underestimation of patients with clinically relevant elevation of inflammatory biomarkers. Additionally, some studies did not exclude or adjust for infection, potentially causing a major bias in the results (10, 12–14). Finally, follow-up varied from 30 days to 5 years (11, 13).

Taken together, even despite the major heterogeneity in outcome measures, length of follow-up, and adjustment for infection, studies consistently showed an increased risk of new vascular events or death in patients with elevated levels of WBC at admission compared to normal levels. Our results support and extend the existing results, by demonstrating an increased risk in a 10-year follow-up period while simultaneously taking competing risks and relevant confounding into account.

### Inflammation in stroke

Increased levels of WBC may be a response to ischemic stroke in the absence of overt infection but can also indicate chronic inflammation, representing a possible underlying disease-causing stroke. Research indicates that chronic inflammation plays an essential role in the initiation and progression of atherosclerotic lesions, potentially leading to ischemic events such as stroke (16). Chronic inflammatory conditions such as rheumatoid arthritis, inflammatory bowel disease, and psoriasis have frequently been associated with an increased risk of stroke (17). Similarly, cancer patients often experience both intrinsic inflammation within their tumors and extrinsic inflammation as a response to the tumor while also facing a heightened risk of stroke (18). The elevated WBC can also be a result of a hyperproliferative state in the bone marrow as seen with chronic myeloid cancers (MPN). MPNs are characterized by elevated blood cell markers and chronic inflammation, and an increased risk of thrombosis (19–22). Notably, in MPNs, elevated levels of WBC are known to be an independent risk factor for thrombosis (23). Thus, the increased levels of WBC and the association with risk of vascular events found in the stroke population could be linked to early stages of these hematological diseases, either as clonal hematopoiesis of indeterminate potential (CHIP) or undiagnosed MPN’s, which have been shown to be massively underdiagnosed chronic blood cancers (24). This is even more pertinent, considering that the most prevalent somatic mutation in MPN—JAK2V617F—is highly prevalent (11.3%) among patients with ischemic stroke either as CHIP or undiagnosed MPN (25). This finding underscores the urgent need for screening for this potent inflammation-mediating mutation, a new risk factor for stroke, in all newly diagnosed stroke patients. This strategy is foreseen to reduce the increased risk of new strokes by much earlier diagnosis and treatment of MPNs.

Targeting the inflammatory pathway with anti-inflammatory drugs may improve the stability of atherosclerotic plaques. A 2020 review highlighted the lack of randomized controlled trials investigating the effects of AID in patients with prior stroke or transient ischemic attack (TIA) and their impact on the risk of vascular events (26). This gap in the literature leaves uncertainties about the potential effectiveness of anti-inflammatory drugs. Currently, the results of the RCT CONVINCE study, which examines the outcomes of colchicine 0.5 mg/day, are pending (27). Further trials are warranted to evaluate the use of anti-inflammatory drugs in stroke patients when considering the demonstrated reduction in recurrent cardiovascular events in patients with previous myocardial infarction (28).

### Limitations and strengths

A strength of this study is the thorough review of each patient’s electronic medical records, resulting in little missing data, as the Danish record system is known for its high level of granularity (29). This manual review of patient records identifies diagnosis even if it has been inaccurately or not coded at all. Statistically, competing risk analysis was used to assess new vascular events in both the Aahlen Johansen analysis and the Cox proportional model, resulting in more accurate estimates of hazard rates. Finally, the current study provided a follow-up of 10 years, which, to the best of our knowledge, is the longest follow-up reported in this field of research.

We enrolled 395 patients, a relatively small sample size compared to prior registry studies. The decision to conduct a manual review, which is a resource-demanding approach, resulted in this smaller sample size. One of our inclusion criteria was symptom onset within 48 h but time to treatment was not differentiated in our study population. As the efficacy of treatment declines rapidly after stroke onset, the risk of impairment increases (30). Delayed revascularization causes increased inflammation and parenchymal damage ultimately resulting in increased mortality and morbidity (31). Therefore, subdividing patients into groups of “time to hospitalization,” may potentially increase the homogeneity of this study; however, the study was too underpowered to allow this stratification. Furthermore, we did not account for the variation in the inflammatory response among different etiological subtypes, which in conjunction with their diverse effects on mortality may introduce potential bias in our findings (32). Patients’ laboratory results and imaging were systematically reviewed to detect DVT, PE, AMI, and ischemic stroke; however, we were not able to systematically screen for other types of vascular events, such as retinal artery occlusion, peripheral arterial thrombosis, and gastrointestinal thrombosis. This could have led to an underestimation of vascular events. We sought to mitigate this issue by reviewing all discharge letters. Most importantly, cancer, rheumatological, and hematological diseases were not adjusted for as confounders. As previously described patients with cancer, both hematological and non-hematological diseases have increased systemic inflammation as well as increased risk of stroke (18, 21). Therefore, elevated risks of new vascular events or death in patients with high levels of WBC may be explained by the presence of these conditions, potentially causing bias toward higher rates of new vascular events and death. Our study did not consider stroke severity, stroke treatment, and premorbid function, all of which can potentially influence the rates of outcome. This omission may have introduced bias to our results (33–36). This study shows that elevated WBC at admission not only increases intermediate and short-term risk of new vascular events and death but also increases long-term risk. This suggests that elevated inflammation markers in patients at baseline may not merely reflect acute inflammation but may also represent chronic inflammation. This should incite further investigations of possible underlying diseases causing elevated WBC to distinguish between acute inflammation and possible chronic inflammation. Monitoring the differential blood cell count and if needed treating the cause of elevated inflammatory biomarkers after ischemic stroke may lower the incidence of new vascular events and death.

## Conclusion

This retrospective observational study assessed long-term increased risk in ischemic stroke patients with elevated levels of WBC at admission. We demonstrated that elevated levels of WBC in patients with acute ischemic stroke are at increased risk of death and new vascular events, even when adjusting for infection. The interpretation of our results is limited by the absence of adjustment to premorbid functional status, stroke severity, and stroke treatment. Acknowledging the limitations, our study extends important knowledge to the area of inflammation as a risk factor in ischemic stroke patients. Our study stresses the need for follow-up of ischemic stroke patients with elevated levels of WBC to explore the underlying reason herein the prevalent inflammation-mediating and stroke-promoting JAK2V617F mutation.

## Data availability statement

The raw data supporting the conclusions of this article will be made available by the authors, without undue reservation.

## Ethics statement

Ethical approval was not required for the study involving humans in accordance with the local legislation and institutional requirements. Written informed consent to participate in this study was not required from the participants or the participants’ legal guardians/next of kin in accordance with the national legislation and the institutional requirements.

## Author contributions

TV, MK, and TW designed the study. TV collected, analyzed, and interpreted the data. TV wrote the manuscript with MK, HH, and TW contributing to revision and improvement. All authors contributed to the article and approved the submitted version.

## Conflict of interest

The authors declare that the research was conducted in the absence of any commercial or financial relationships that could be construed as a potential conflict of interest.

## Publisher’s note

All claims expressed in this article are solely those of the authors and do not necessarily represent those of their affiliated organizations, or those of the publisher, the editors and the reviewers. Any product that may be evaluated in this article, or claim that may be made by its manufacturer, is not guaranteed or endorsed by the publisher.
